# Highly Efficient Blue‐Emitting CsPbBr_3_ Perovskite Nanocrystals through Neodymium Doping

**DOI:** 10.1002/advs.202001698

**Published:** 2020-09-03

**Authors:** Yujun Xie, Bo Peng, Ivona Bravić, Yan Yu, Yurong Dong, Rongqing Liang, Qiongrong Ou, Bartomeu Monserrat, Shuyu Zhang

**Affiliations:** ^1^ Institute for Electric Light Sources Department of Light Sources and Illuminating Engineering and Academy for Engineering and Technology Fudan University Shanghai 200433 P. R. China; ^2^ Cavendish Laboratory University of Cambridge J. J. Thomson Avenue Cambridge CB3 0HE UK; ^3^ Department of Materials Science and Metallurgy University of Cambridge 27 Charles Babbage Road Cambridge CB3 0FS UK

**Keywords:** bandgap tunability, blue emission, dopant‐induced electronic change, neodymium doping, perovskite nanocrystals

## Abstract

Colloidal CsPbX_3_ (X = Br, Cl, and I) perovskite nanocrystals exhibit tunable bandgaps over the entire visible spectrum and high photoluminescence quantum yields in the green and red regions. However, the lack of highly efficient blue‐emitting perovskite nanocrystals limits their development for optoelectronic applications. Herein, neodymium (III) (Nd^3+^) doped CsPbBr_3_ nanocrystals are prepared through the ligand‐assisted reprecipitation method at room temperature with tunable photoemission from green to deep blue. A blue‐emitting nanocrystal with a central wavelength at 459 nm, an exceptionally high photoluminescence quantum yield of 90%, and a spectral width of 19 nm is achieved. First principles calculations reveal that the increase in photoluminescence quantum yield upon doping is driven by an enhancement of the exciton binding energy due to increased electron and hole effective masses and an increase in oscillator strength due to shortening of the Pb—Br bond. Putting these results together, an all‐perovskite white light‐emitting diode is successfully fabricated, demonstrating that B‐site composition engineering is a reliable strategy to further exploit the perovskite family for wider optoelectronic applications.

## Graphical abstract

Narrowband blue‐emitting CsPbBr_3_ perovskite nanocrystals with a photoluminescence quantum yield of 90% are achieved by B‐site doping of neodymium ions. The doping concentration can tune the emission spectrum in a controlled manner. First principles calculations reveal that dopant‐induced electronic changes dominate the bandgap tunability and the high quantum yield is associated with enhanced exciton binding energy and oscillator strength.

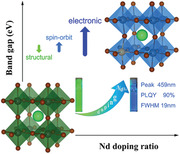

## 1 Introduction

All‐inorganic cesium lead halide perovskite CsPbX_3_ (X = Br, Cl, and I) nanocrystals (NCs) have experienced rapid development since the first report in 2015.^[^ ^1^ ^]^ Due to their intriguing properties such as the high photoluminescence quantum yields (PLQYs) and narrowband single‐peak emission profiles, the flexibility of composition and associated bandgap engineering, and the facile procedures for material synthesis, CsPbX_3_ NCs hold great potential for applications in areas including light‐emitting diodes,^[^ ^2^, ^3^ ^]^ lasers,^[^ ^4^, ^5^ ^]^ solar cells,^[^ ^6^, ^7^ ^]^ and photodetectors.^[^ ^8^ ^]^ In particular, NCs can be used as color‐conversion phosphors for white light‐emitting diodes (WLEDs) and exhibit a wide color gamut coverage.^[^ ^9^ ^]^ One outstanding challenge of all CsPbX_3_ NCs is the toxicity of lead. Additionally, the blue part of the spectrum is typically obtained from chloride‐based perovskite NCs, which presently suffer from low stability and/or relatively low PLQYs, thus limiting the incorporation of perovskite NCs into devices.^[^ ^10^ ^]^

One promising solution to addressing these challenges is to fully or partially replace Pb^2+^ ions by B‐site dopants.^[^ ^11^, ^12^ ^]^ The doping ions do not only reduce lead toxicity but can also enhance both thermal and phase stability of CsPbX_3_ NCs by approaching an optimized Goldschmidt's tolerance factor.^[^ ^13^ ^]^ B‐site cations also play a critical role in determining the electronic band structure of perovskites and consequently their emission properties. Recent studies have demonstrated successful B‐site doping using alkaline‐earth metal ions (Mg^2+^, Ba^2+^, Sr^2+^),^[^ ^14^, ^15^, ^16^, ^17^ ^]^ transition metal ions (Cu^2+^, Cd^2+^, Ag^+^, Zn^2+^),^[^ ^18^, ^19^, ^20^, ^21^ ^]^ metalloid ions (Sn^2+^, Bi^3+^),^[^ ^22^, ^23^, ^24^ ^]^ and lanthanide ions (Ce^3+^, Tb^3+^, Yb^3+^).^[^ ^25^, ^26^, ^27^ ^]^ Despite these advantages, dopants may also lead to negative effects on the properties of NCs. Begum et al. have found that Bi^3+^ doping can cause significant emission quenching due to the appearance of defect states in the bandgap that act as trapping and scattering centers.^[^ ^23^ ^]^ Dual emission is another commonly observed feature with dopants like Mn^2+^, Yb^3+^, Er^3+^, and Eu^3+^, and it arises from energy transfer from the perovskite host to the dopant guest, with the narrowband single‐peak emission of pristine NCs being inevitably compromised.^[^ ^6^, ^28^, ^29^, ^30^ ^]^ Interestingly, van der Stam et al. have demonstrated a successful photoluminescence (PL) blueshift without additional emission peaks by partially postexchanging Pb^2+^ with Sn^2+^, Cd^2+^, or Zn^2+^.^[^ ^22^ ^]^ Liu et al. have also reported Al^3+^‐doped CsPbBr_3_ NCs with a deep blue single‐peak emission at 456 nm.^[^ ^31^ ^]^ These studies show that dual emission can be avoided with adequate dopants, but the PLQYs of the aforementioned blue‐emitting NCs are still unsatisfying.

In this work, we synthesize highly efficient blue‐emitting perovskite NCs by integrating Nd^3+^ into CsPbBr_3_ NCs as B‐site dopants. We demonstrate that, as the doping concentration increases, the narrowband single‐peak emission of CsPbBr_3_:*x*Nd^3+^ NCs (where *x* is the atomic doping ratio *x* = Nd/(Nd + Pb)) can be fine‐tuned from green to deep blue with a PLQY value in the range of 75–90% and a spectral width below 25 nm. At *x *= 7.2%, we obtain blue emission at 459 nm with a PLQY of 90% and a spectral width of only 19 nm. Using first principles calculations, we find that the bandgap tunability results from breaking translational symmetry with B‐site substitution, which simultaneously stabilizes the valence band maximum (VBM) and destabilizes the conduction band minimum (CBM). This purely electronic mechanism contrasts with the picture discussed for other dopants, in which structural changes are proposed to dominate. We also show that the increase in PLQY is a result of the increased exciton binding energy due to the flattening of the valence and conduction bands upon doping and the enhanced exciton oscillator strength due to the lattice contraction induced by Nd doping. Equipped with the highly efficient blue‐emitting perovskite NCs, we fabricate an all‐perovskite phosphor‐based WLED using blue‐emitting CsPbBr_3_:*x*Nd^3+^ NCs (*x *= 7.2%) NCs, green‐emitting pristine CsPbBr_3_ NCs and red‐emitting CsPbBr_1.2_I_1.8_ NCs and achieve a National Television System Committee (NTSC) value of 122% and Rec. 2020 of 92%.

## 2 Results and Discussion

**Figure 1:**
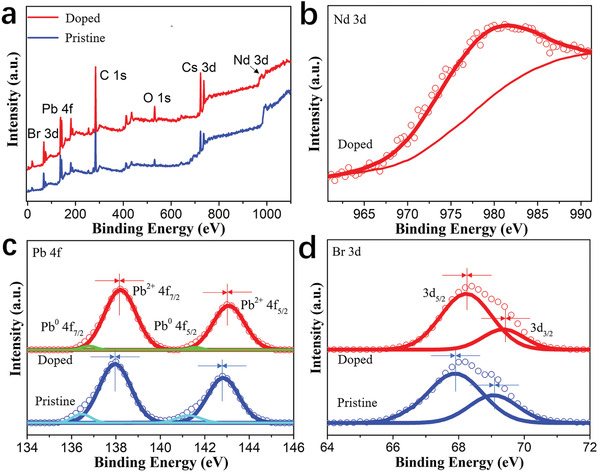
a) XPS spectra for the films of CsPbBr_3_:*x*Nd^3+^ (*x* = 7.2%) NCs and pristine CsPbBr_3_ NCs. High‐resolution XPS spectra corresponding to b) Nd^3+^ 3d, c) Pb^2+^ 4f, and d) Br^−^ 3d, respectively. The hollow circular symbol represents the raw data and the solid curve represents the corresponding fitting curve.

The Nd^3+^‐doped CsPbBr_3_ NCs are prepared through the modified ligand‐assisted reprecipitation (LARP) method (see the Experimental Section for details).^[^ ^9^, ^32^ ^]^ Simply, PbBr_2_, CsBr, and dopants NdBr_3_ are codissolved in *N*,*N*‐dimethylformamide (DMF) as the precursor solution, and oleic acid (OA) and oleylamine (OLAm) are mixed to stabilize it. Then, the precursor solution is added into toluene under vigorous stirring to form perovskite NCs through crystallization. After synthesizing the doped NCs with different Nd concentrations, we test whether Nd^3+^ ions have been successfully incorporated into the host lattice by replacing some of the Pb^2+^ ions. It has been reported that lanthanide ions tend to take over the Pb^2+^ sites to achieve the lowest formation energy in both Pb‐poor and Pb‐rich conditions.^[^ ^28^ ^]^ To verify this so‐called B‐site doping of Nd^3+^, we perform X‐ray photoelectron spectroscopy (XPS) measurements on films of Nd^3+^‐doped CsPbBr_3_ NCs and pristine CsPbBr_3_ NCs (see **Figure** **1**a) and we see that the XPS peaks of Cs 3d, Pb 4f, Br 3d, as well as O 1s and C 1s, can be found in both cases. The additional peak in Nd^3+^‐doped CsPbBr_3_ NCs at 980.2 eV can be assigned to the Nd 3d signal, indicating the presence of Nd in the films (see Figure 1b). Furthermore, by means of XPS peak‐differentiation‐imitating analysis, the high‐resolution spectrum of Pb 4f for the pristine NCs consists of two major peaks of Pb^2+^ 4f_5/2_ at 142.8 eV and Pb^2+^ 4f_7/2_ at 138.0 eV and two shoulder peaks at 141.2 and 136.4 eV associated with metallic Pb^0^ (see Figure 1c).^[^ ^33^ ^]^ For Nd^3+^‐doped CsPbBr_3_ NCs, the Pb^0^ peaks are suppressed and the Pb^2+^ 4f peaks move to higher binding energy. This trend is also observed for the Br^−^ 3d_3/2_ and 3d_5/2_ peaks (see Figure 1d) but not for Cs^+^ 3d_3/2_ and 3d_5/2_ peaks (see Figure S1, Supporting Information), which suggests stronger Pb‐Br interactions^[^ ^31^ ^]^ caused by lattice contraction after Nd^3+^ incorporation. As a result, we conclude that Nd^3+^ ions successfully enter the lattice of the CsPbBr_3_ perovskite host and take over a fraction of the Pb^2+^ sites.

**Figure 2:**
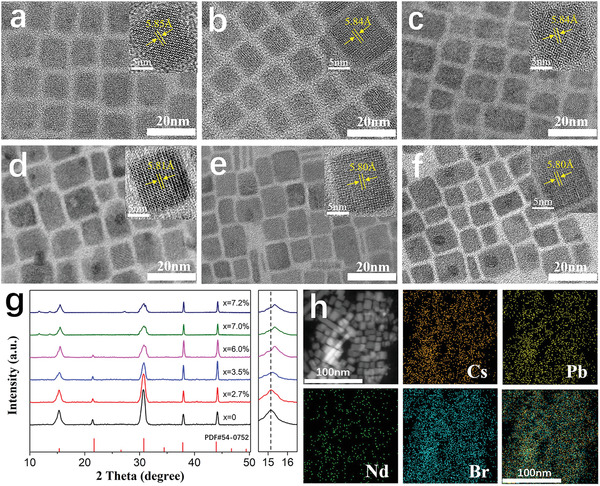
TEM images of Nd^3+^‐doped CsPbBr_3_ NCs with a Nd/(Nd + Pb) doping ratio of a) *x *= 0, b) *x* = 2.7%, c) *x* = 3.5%, d) *x* = 6.0%, e) *x* = 7.0%, and f) *x* = 7.2%, and their corresponding high‐resolution transmission electron microscopy (HRTEM) images are shown in the insets. g) XRD patterns of all CsPbBr_3_:*x*Nd^3+^ samples. h) The HAADF‐STEM image and elemental mappings of Cs, Pb, Nd, and Br elements in the CsPbBr_3_:*x*Nd^3+^ (*x* = 7.2%) NCs the last frame shows the overlap of the elemental mapping images.

We next examine the structural effects in the presence of Nd dopant. **Figure** **2**a–f shows the transmission electron microscope (TEM) images of Nd^3+^‐doped CsPbBr_3_ NCs with different doping ratios, indicating the heterovalent doping of Nd^3+^ ions lowers the uniformity in size and shape of the particles. The average size of NCs decreases as the Nd^3+^ doping concentration increases (see Figure S2, Supporting Information) because the larger amount of halide ions constrains the further growth of NCs.^[^ ^21^ ^]^ The black spots observed in TEM images are supposed to be the reduction of Pb^2+^ ions to metallic Pb^0^ since the perovskite NCs are exposed to high‐energy incident electron beam for a long time during the measurements.^[^ ^34^, ^35^ ^]^ The interplanar distances of the (100) planes of Nd^3+^‐doped CsPbBr_3_ NCs also decrease at higher doping concentration, from 5.85 Å for pristine CsPbBr_3_ NCs to 5.80 Å for CsPbBr_3_:*x*Nd^3+^ (*x *= 7.2%) NCs (see insets of Figure 2a–f). This trend is verified by our first principles calculations: during the structural relaxation of 7.4% Nd^3+^‐doped CsPbBr_3_, the lattice constant decreases from 5.87 to 5.82 Å (see Table S1, Supporting Information). The lattice contraction is due to the incorporation of smaller Nd^3+^ (0.98 Å) replacing larger Pb^2+^ (1.19 Å) in the [BX_6_]^4−^ octahedron. Our first principles calculations indicate that the larger electronegativity of Pb compared to Nd also contributes to the lattice contraction, as electrons transfer from Nd to the neighboring Pb—Br pairs, leading to an enhancement of the Pb—Br bond strength which reduces its bond length.

The X‐ray diffraction (XRD) patterns shown in Figure 2g indicate that the perovskite structures are formed in both doped and pristine NCs. The main diffraction peaks appear at 15.1°, 21.5°, 30.7°, 38.0°, and 44.2°, which are assigned to the (100), (110), (200), (211), and (220) planes, respectively, indexed by a cubic crystal system (Joint Committee on Powder Diffraction Standards [JCPDS] Portable Document Format (PDF) #54‐0752). These diffraction peaks shift to higher angles with increasing Nd^3+^ concentration, which confirms the macroscopic lattice contraction.

The increased doping concentration also leads to peak broadening and splitting at 30.7°, which indicates a symmetry‐lowering tilt and distortion of the [BX_6_]^4−^ octahedron.^[^ ^17^, ^29^ ^]^ Such distortions are also predicted by our first principles calculations, but they are below 1.5°. It has been reported that doping can induce structural evolution, especially when the size mismatch between the dopant and substituted atoms is large,^[^ ^29^, ^31^, ^36^ ^]^ but the calculated small distortion here indicates the lattice contraction and octahedral tilting are not strong enough to induce a distinct structural change. Furthermore, the additional weak peaks observed in the range of 15° to 20° and 25° to 30° at *x* > 7.0% are attributed to an impurity phase. To determine the nature of the impurity phase, the products from mixed CsBr and NdBr_3_ precursors without lead participation are prepared (see Figure S3, Supporting Information). No PL emission is observed but the asterisk marked peaks in the XRD pattern reveal the same unindexed phase observed in CsPbBr_3_:*x*Nd^3+^ (*x* = 7.0% and 7.2%) NCs.

The high‐angle annular dark‐field scanning transmission microscopy (HAADF‐STEM) image of Nd^3+^‐doped CsPbBr_3_ NCs is shown in Figure 2h and the corresponding energy dispersive X‐ray spectroscopy (EDS) mapping images of Cs, Pb, Br, and Nd which overlap well with the HAADF‐STEM image indicate the existence of Nd^3+^ in NCs. The atomic percentage of dopants (see Figure S4, Supporting Information) increases with the amount of NdBr_3_ added into the mixed precursor. The quantitative Nd‐doping ratio of CsPbBr_3_:*x*Nd^3+^ NCs is less than 8% measured by inductively coupled plasma mass‐spectrometry (ICP‐MS, see Table S2, Supporting Information), which means the doping level of Nd^3+^ is relatively low.

**Figure 3:**
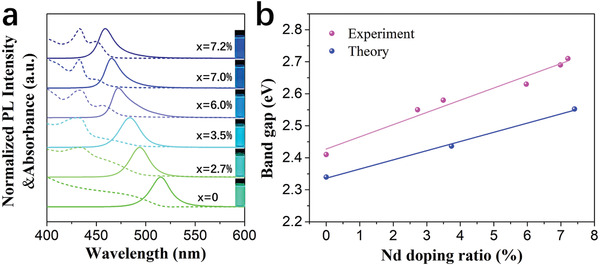
a) Normalized PL and absorbance spectra of CsPbBr_3_:*x*Nd^3+^ NC colloidal solutions with different Nd/(Nd + Pb) doping ratios: PL emission (solid line, excitation at 365 nm) and UV‐visible absorbance spectra (dashed line). The inset shows the corresponding images of luminescent CsPbBr_3_:*x*Nd^3+^ NC colloidal solutions under UV excitation. b) Broadening of the bandgap with the increasing Nd doping ratio.

**Figure** **3**a shows the normalized PL and absorbance spectra of doped CsPbBr_3_ NC solutions. As the doping ratio increases, both the absorption onset and the PL peak exhibit a continuous blueshift. This behavior is quantified in the Tauc plots of (*αhν*)^2^ versus photon energy (*hν*) based on the absorption spectra (see Figure S5, Supporting Information), showing that the bandgap of Nd^3+^‐doped CsPbBr_3_ NCs increases with increasing Nd^3+^ doping ratio. We show that the B‐site doping of Nd^3+^ ions in the host CsPbBr_3_ NCs leads to an apparent blueshift from 515 to 459 nm with increased PLQY. In contrast, for those using other dopants, the PL peak only changes slightly or even remains the same at much higher doping concentration, e.g., for 23% Mg^2+^, 35.5–43% Cd^2+^, and 13.8% Ni^2+^ doped CsPbCl_3_, the PL peak remains at 406 nm;^[^ ^10^, ^14^, ^19^ ^]^ for 23% Cu^2+^ doped CsPbBr_3_, the PL peak only experiences a blueshift from 517 to 499 nm.^[^ ^18^ ^]^ In these previous works, the B‐cation doping alone cannot tune the PL peak position effectively. Here we show tunable emission as a result of B‐site doping of Nd^3+^ at a much lower doping concentration. We also notice that the increasing strain field resulting from the progressive lattice contraction may halt the further doping of Nd^3+^ ions, making the doping reaction self‐limited.^[^ ^22^ ^]^

Using first principles methods, we calculate the bandgap of CsPbBr_3_ as a function of doping concentration (see the Experimental Section), and Figure 3b shows that the resulting bandgap blueshift is in good agreement with the experimental trend. In our calculations we include all of structural effects, many‐body effects, spin–orbit coupling, and thermal effects using state‐of‐the‐art many‐body *GW* calculations and also include a correction to account for thermal effects. It is the first time, as far as we are aware, in which all these effects are incorporated, making our calculations state‐of‐the‐art. Despite including all these terms, there is still a disagreement in the value of the bandgap compared to experiment, and we attribute it to the remaining difference between the computational model and experiment, which is due to quantum confinement effects in NCs that can be significantly over 0.05 eV even for pristine CsPbBr_3_.^[^ ^37^ ^]^ This last contribution is impossible to include in our calculations because the system sizes involved are prohibitively large. Nonetheless, despite this residual difference for the absolute value of the bandgap between theory and experiment arising from quantum confinement, we point out that we are interested in studying the trends in the bandgap with doping concentration, and trends are more robust than absolute values.

**Figure 4:**
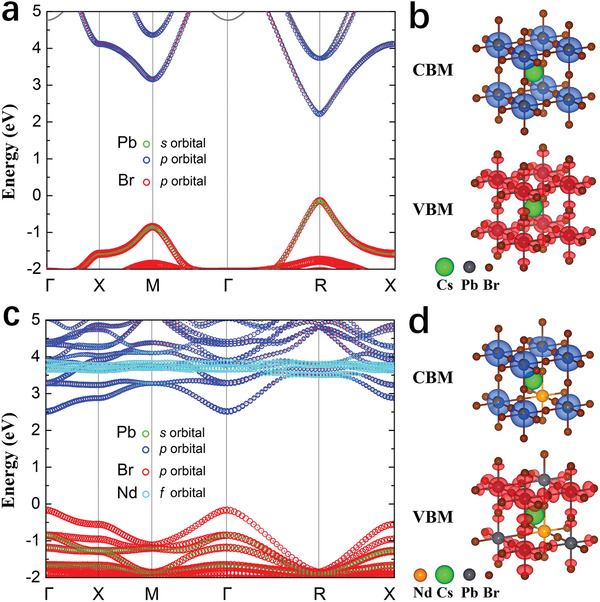
a) Calculated band structure of pristine CsPbBr_3_. The orbital characters are plotted as a fat‐band structure showing Pb 6s, 6p, and Br 4p orbitals. b) Partial charge densities of the VBM and the CBM of pristine CsPbBr_3_. c) Calculated band structure of CsPbBr_3_:*x*Nd^3+^ (*x* = 12.5%). The orbital characters are plotted as a fat‐band structure showing Pb 6s, 6p, and Br 4p orbitals as well as Nd 5d orbitals. d) Partial charge densities of the VBM and the CBM of Nd^3+^‐doped CsPbBr_3_.

To rationalize the effect of B‐site doping of Nd^3+^ ions on the optical properties of CsPbBr_3_, we calculate the electronic structure of bulk pristine and Nd^3+^‐doped CsPbBr_3_. The calculated band structure of pristine CsPbBr_3_ is shown in **Figure** **4**a. We obtain a direct bandgap at the R‐point of the Brillouin zone (BZ) that has a value of 2.34 ± 0.06 eV, which agrees well with the experimental photoluminescence peak at 2.41 eV when considering the weak quantum‐confinement effect present in the NCs (see Table S3 and Figure S5a, Supporting Information). The VBM results from the antibonding interaction between Pb 6s and Br 5p orbitals, leading to a destabilized state with respect to the isolated Pb 6s and Br 5p states. This interaction results in an isotropic delocalized state along the Pb—Br vertices of the lattice with nodes on the Pb—Br bond (see Figure 4b). The CBM consists of empty Pb 6p states that hybridize to build a bonding and stabilized conduction band minimum with respect to the atomic orbitals (see Figure 4b). Our results are consistent with previous calculations.^[^ ^38^, ^39^, ^40^ ^]^

The band structure calculations with Nd dopants at Pb sites show that the bandgap increases with increasing doping concentration. Figure 4c depicts the electronic structure of CsPbBr_3_:*x*Nd^3+^ (*x *= 12.5%), in which the bandgap increases from 2.34 to 2.59 eV. When Pb is replaced with Nd, the formation of the valence band described above is perturbed, giving a band that mostly resembles the energetically lower lying isolated Br 5p orbitals. This is shown in Figure 4d by the VBM with dominant Br contributions, by the change in dispersiveness toward a more localized state compared to the pristine case, and by the change in the spatial charge distribution of the VBM. Similarly, the periodic overlap of the CBM Pb 6p orbitals is perturbed, which leads to a destabilization of the conduction band toward the atomic 6p levels. The simultaneous stabilization of the VBM and destabilization of the CBM results in the observed bandgap increase (see Figure S6, Supporting Information).

The key point of our microscopic mechanism is that, different from previous works that attribute the doping‐induced blueshift to structural effects,^[^ ^14^, ^18^, ^22^, ^41^ ^]^ we find that structural effects instead lead to a slight redshift. After a systematic theoretical study on the individual contributions as well as the interplay between electronic effects, spin–orbit coupling, and structural effects including lattice contraction and octahedral distortion, we demonstrate that the blueshift is dominated by electronic effects (see Figure S7, Supporting Information). We observe that the blueshift appears even when the replacement of Pb by Nd is not followed by a structural relaxation of the defective supercell. In fact, after the structural relaxation with Nd dopants, the volume of the cell decreases, the Pb—Br bond lengths decrease, and the [BX_6_]^4−^ octahedra slightly tilt, and these combined effects result in a relative redshift of the bands compared to the unrelaxed supercell. It is true that previous works have claimed that the blueshift is attributed to lattice contraction.^[^ ^14^, ^18^, ^22^ ^]^ However, detailed experimental studies also provide clear evidence that lattice contraction leads to a redshift while octahedral tilting leads to blueshift.^[^ ^42^, ^43^, ^44^ ^]^ To understand the redshift induced by structural changes, we note that when Pb is exchanged with Nd, lattice contraction is caused by the charge redistribution from the Nd toward the neighboring Pb—Br pairs as Nd has smaller electronegativity than Pb. With decreasing Pb—Br bond length, the antibonding interaction between the Pb 6s and Br 5p states building the VBM increases, which in turn shifts the band toward higher energies. As the conduction band is only made of Pb 6p states the CBM remains mostly invariant upon changes in the Pb—Br bond length. This relative shift of the valence band maximum is responsible for the structurally induced redshift, which competes with the larger electronically induced blueshift, leading to an overall blueshift. Finally, we note that spin–orbit coupling enhances the blueshift with increasing Nd concentrations, as previously found with other dopants,^[^ ^45^ ^]^ but we remark that it is not the dominant effect in driving the blueshift. As an example, at 12.5% doping, the electronically induced blueshift is 0.24 eV, structural relaxation leads to a redshift of 0.08 eV, and spin–orbit coupling results in a blueshift of 0.09 eV, leading to an overall blueshift of 0.25 eV. This microscopic understanding provides new insights to guide further experimental studies of composition engineering in colloidal CsPbX_3_ NCs and provides a framework for further study of doping effects in other material systems.

The intrinsic PL peak of pristine CsPbBr_3_ NCs sits at 515 nm at room temperature and can be gradually tuned to 459 nm when the *x* value reaches 7.2%, while no additional dopant‐related peaks appear in the visible and near infrared spectral range (see Figure 3a). Nd^3+^‐doped CsPbBr_3_ NCs exhibit PLQY values in the range from 75% to 90% and a spectral width below 25 nm (see Table S4, Supporting Information). For CsPbBr_3_:*x*Nd^3+^ (*x* = 7.2%) NCs, the PLQY value reaches 90% and the spectral width is only 19 nm. To the best of our knowledge, the PLQY value of CsPbBr_3_:*x*Nd^3+^ (*x* = 7.2%) NCs is more than double that of the values of previously reported blue‐emitting chlorine‐based mixed halide CsPbX_3_ and MAPbX_3_ NCs^[^ ^9^, ^46^ ^]^ and also surpasses the reported values of blue‐emitting A/B‐site doped perovskite NCs in prior work^[^ ^18^, ^22^, ^31^, ^47^ ^]^ (see Table S5, Supporting Information).

**Figure 5:**
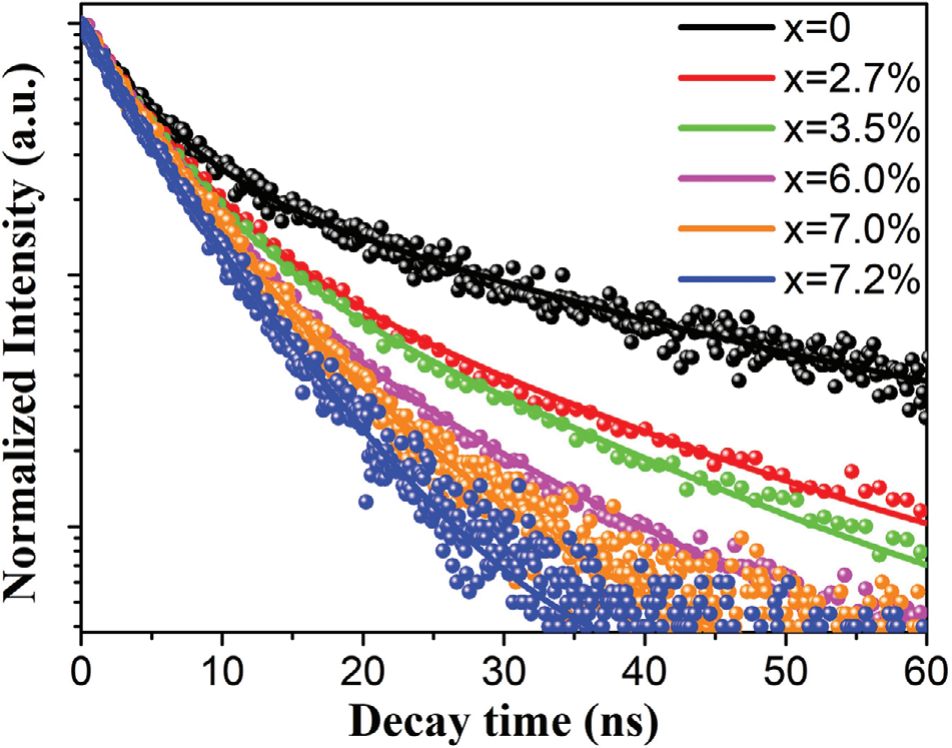
The decay curves of time‐resolved photoluminescence of the CsPbBr_3_:*x*Nd^3+^ NCs solutions.

To investigate the radiative mechanism, we measure time‐resolved photoluminescence curves of Nd^3+^‐doped CsPbBr_3_ NC solutions (see **Figure** **5**). All the curves are fitted by a biexponential decay model with a fast and a slow decay components (see Table S6, Supporting Information). As the *x* value increases from 0% to 7.2%, the average lifetime (*τ*_ave_) decreases from 14.11 to 5.27 ns. The radiative decay rates (*k*_r_) and the nonradiative decay rates (*k*_nr_) are also listed in Table S6 in the Supporting Information. The *k*_r_ value increases as the doping ratio increases, so that the *k*_r_ value of CsPbBr_3_:*x*Nd^3+^ (*x* = 7.2%) NCs are approximately three times larger than that of the pristine NCs. By contrast, the *k*_nr_ value first increases slightly due to the increasing number of defects caused by the heterovalent doping,^[^ ^48^ ^]^ but then gradually decreases when the *x* value is over 3.5%, which could be ascribed to the reduction of surface metallic Pb^0^ defect states as shown in the XPS results (see Figure 1c).^[^ ^34^ ^]^ It is believed that the introduction of shallow energy levels and curing of the intrinsic structural disorder are the main reasons of improved PLQY in Cd^2+^ and Mg^2+^ doped CsPbCl_3_.^[^ ^14^, ^19^, ^49^ ^]^ We find that the radiative recombination rate *k*_r_ exhibits a more significant change according to the PL decay while the nonradiative recombination rate *k*_nr_ only experiences a slight change. Therefore the enhancement of *k*_r_ is the main factor promoting the luminescence quantum efficiency, rather than the suppression of *k*_nr_ that is related to defect states. This is consistent with the fact that CsPbBr_3_ is found to be highly defect‐tolerant in terms of both PL position and PLQY.^[^ ^49^, ^50^, ^51^ ^]^ Different from CsPbCl_3_ with weak luminescence (≈3%) due to structural defects, the PLQY for pristine CsPbBr_3_ is already very high (≈81%), indicating small effects of structural defects. Our result shows that the dominant mechanism is the increased radiative recombination and we perform first principles calculations on the defect‐free system to understand how *k*_r_ increases with doping.

We identify two microscopic mechanisms to explain the increase of *k*_r_ with increasing doping observed experimentally. The first mechanism is associated with the flattening of the valence and conduction bands upon doping (see Figure 4c), increasing the electron and hole effective masses. According to the Wannier–Mott model, larger effective masses lead to stronger spatial confinement of the exciton and to an increase in its binding energy (see Table S7 and Figure S8, Supporting Information). The second mechanism is associated with the doping‐driven decrease in the Pb—Br bond length. The lowest energy bright exciton is dominated by the electron–hole pair at the band edges, implying that photoexcitation leads to a transfer of an electron along the Pb—Br bond toward the Pb ion, while the hole remains delocalized over both elements. This suggests that decreasing the Pb—Br bond length pushes the system toward a brighter state, leading to an increase in the *k*_r_. To demonstrate the lattice‐contraction mechanism, we conduct exciton oscillator strength calculations for the pristine system, but using the smaller primitive cell parameters that correspond to those of the 12.5% Nd‐doped system. As expected, we find that the oscillator strength of the first bright exciton increases by 67.2% with respect to the fully relaxed pristine perovskite, proving that lattice contraction is one of the key factors driving the increase in *k*_r_. This is due to the increase in the overlap between the wave function of electron and hole states that form the exciton. Based on first principles calculations, we show that the PLQY of defect‐free CsPbBr_3_ can be further enhanced by lattice contraction. Such lattice contractions have also been observed in Mg^2+^ and Cu^2+^ doped systems with dramatic enhancements of the PLQY.^[^ ^14^, ^18^ ^]^ Thus our explanation could be a universal mechanism of enhanced PLQYs in doped CsPbBr_3_.

We next investigate the stability of the Nd‐doped NCs. The atomic radius of Nd^3+^ (*r*_B_ = 0.98 Å) is smaller than that of Pb^2+^ (*r*_B_ = 1.19 Å), leading to the increase of the tolerance factor^[^ ^52^ ^]^ and suggesting a possible improvement in stability. To confirm this, a thermal cycling experiment is performed for pristine NC and Nd^3+^‐doped CsPbBr_3_ NC solutions and the results are shown in Figure S9 in the Supporting Information. As expected, the doped NCs exhibit better thermal stability than the pristine ones. 90% of the initial PL intensity is preserved for doped NCs but only 55% is preserved for pristine ones. Moreover, the Nd^3+^ dopants also inhibit any PL peak shift during the heating process, making the blue‐emitting NCs an ideal phosphor. The air and photostability are also investigated for Nd^3+^ doped system. Both pristine and doped colloidal CsPbBr_3_ NCs retain about 90% of the relative PLQYs after 30 d in ambient conditions. After 8 h, about 60% of the relative PL is preserved for both pristine and doped system under continuous ultraviolet irradiation at a wavelength of 365 nm and with a power density of 141 mW cm^−2^ (see Figure S10, Supporting Information).

**Figure 6:**
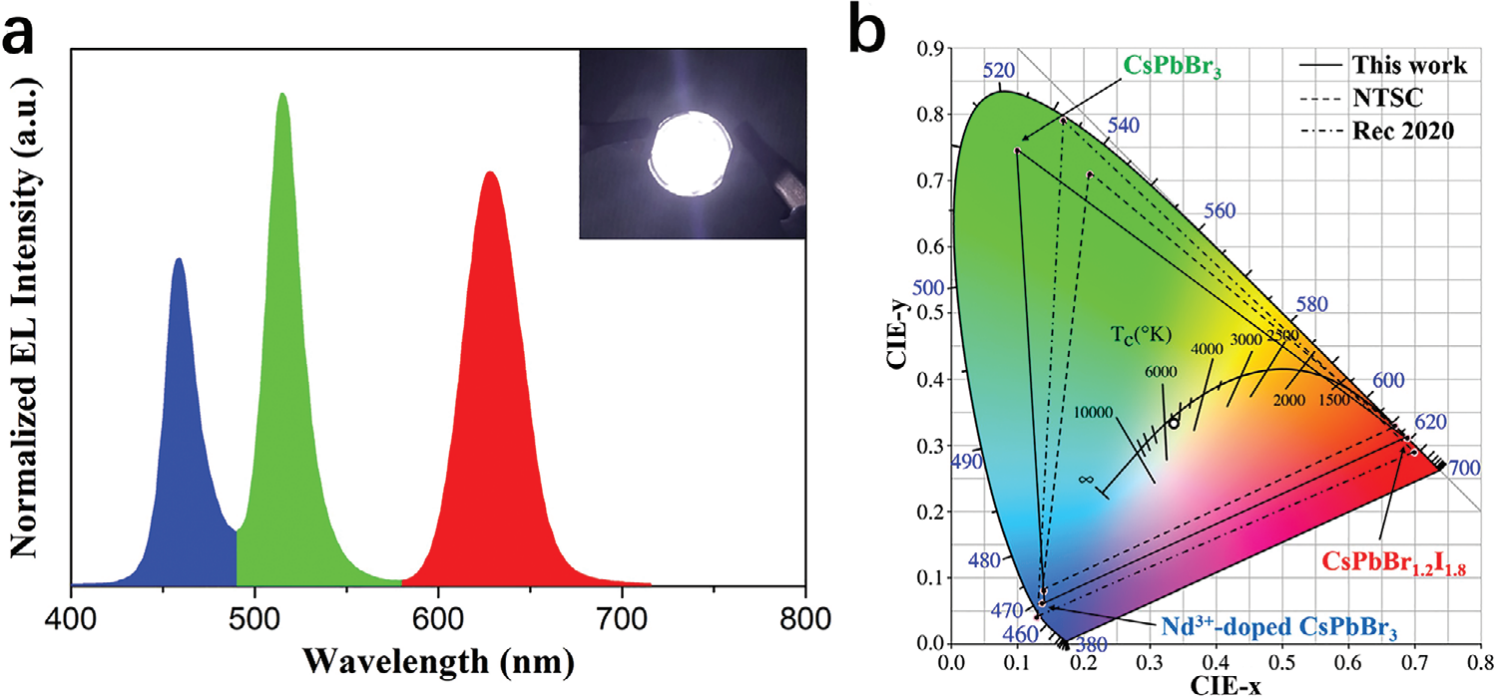
a) Emission spectra of WLED. The inset shows the related photograph of the working WLED. b) Color gamut of the WLED in this work compared to the NTSC Television standard and the Rec. 2020 standard. The white dot shows the CIE color coordinates of the WLED device at (0.34, 0.33).

Since the perovskite NCs are promising candidates to produce saturated colors, we prepare an all‐perovskite phosphor‐based WLED using blue‐emitting Nd^3+^‐doped CsPbBr_3_ NCs, green‐emitting pristine CsPbBr_3_ NCs, and red‐emitting CsPbBr_1.2_I_1.8_ NCs. The perovskite phosphors are excited by a UV LED chip. Unlike the anion fast‐exchange and color‐drifting behavior induced by X‐site halide doping, the mixture of the blue CsPbBr_3_:*x*Nd^3+^ (*x* = 7.2%) NCs and green CsPbBr_3_ NCs shows a superimposed emission with two peaks originating independently from the two different types of NCs (see Figure S11, Supporting Information). A dual‐emission composite film is prepared by encapsulating both CsPbBr_3_ NCs and CsPbBr_3_:*x*Nd^3+^ (*x* = 7.2%) NCs in polymethyl methacrylate (PMMA) and then deposited on the UV‐LED together with a red‐emitting CsPbBr_1.2_I_1.8_/PMMA film. The WLED shows color coordinates optimized at (0.34, 0.33), which corresponds to a cool‐white correlated color temperature of 5310 K. The peak wavelengths of Red‐Green‐Blue (RGB) are 628, 515, and 459 nm and the corresponding RGB Commission Internationale de l'Eclairage (CIE) coordinates are (0.69, 0.31), (0.10, 0.75), and (0.14, 0.06), respectively. Intriguingly, the blue color coordinate is closely approaching the standard specification of Rec. 2020 (0.131, 0.046). **Figure** **6** shows the electroluminescence and color gamut of the fabricated WLED. This all‐perovskite phosphor‐based WLED demonstrates a NTSC value of 122% and Rec. 2020 of 92%, which are excellent parameters for a white backlight.

## 3 Conclusion

In summary, we report, for the first time, successful heterovalent doping of Nd^3+^ in substitution of Pb^2+^ in colloidal CsPbBr_3_ NCs via a facile room‐temperature synthesis method. The doping concentration can be used to tune the emission spectrum from green to blue in a controlled manner. The blue‐emitting CsPbBr_3_:*x*Nd^3+^ (*x* = 7.2%) NCs have a PLQY value of 90% and a spectral width of 19 nm. Using first principles calculations, we demonstrate that the bandgap tunability is mostly driven by dopant‐induced electronic changes, while the increase in PLQY is associated with increased exciton binding energy driven by dopant‐induced electronic changes and enhanced exciton oscillator strength driven by dopant‐induced structural changes. This microscopic understanding opens new possibilities for B‐site composition engineering in colloidal CsPbX_3_ NCs. Integrating RGB perovskite phosphors with a UV‐LED, a WLED with a NTSC gamut value of 122% and Rec. 2020 of 92% is achieved.

## 4 Experimental Section

##### Reagents

All the reagents were used directly without further purification: lead bromide (PbBr_2_, 99.999%, Aladdin), cesium bromide (CsBr, 99.99%, Aladdin), neodymium bromide hexahydrate (NdBr_3_. 6H_2_O, 99.9%, Alfa Aesar), OA (Aladdin), oleylamine (OLAm, Aladdin), and DMF (AR, Sinopharm Chemical Reagent).

##### Synthesis of CsPbX_3_ NCs

The solution preparation of CsPbBr_3_ NCs was adapted from the LARP method.^[^ ^9^, ^32^ ^]^ Briefly, the precursor solution was made by dissolving PbBr_2_ (0.1 mmol) and CsBr (0.1 mmol) in DMF (2.5 mL) and stabilized by adding OA (0.25 mL) and OLAm (0.13 mL). 1 mL precursor solution was added into toluene (10 mL) under vigorous stirring for 1 min at room temperature. As for the CsPbBr_1.2_I_1.8_ NCs, the same procedure was used except that the precursors were PbBr_2_ (0.04 mmol), PbI_2_ (0.06 mmol), CsBr (0.04 mmol), and CsI (0.06 mmol). All steps were carried out under ambient conditions.

##### Synthesis of Nd^3+^‐Doped CsPbBr_3_ NCs

The solution of Nd^3+^‐doped CsPbBr_3_ NCs was prepared by mixing a specific molar quantity of NdBr_3_ in the precursor solution without changing the other operation procedures. For instance, the precursor solution of CsPbBr_3_:*x*Nd^3+^ (*x *= 7.2%) NCs was prepared by dissolving NdBr_3_ (0.15 mmol), PbBr_2_ (0.1 mmol), and CsBr (0.1 mmol) in DMF (2.5 mL) and stabilized by adding OA (0.25 mL) and OLAm (0.13 mL).

##### Purification

The crude samples in toluene were separated by centrifugation at 10 000 rpm for 10 min. The supernatant with strong luminescence was preserved and the precipitate was discarded (pristine and aggregated NCs). Then, to remove the unreacted component and byproducts, methyl acetate was adopted to assist precipitation of the doped NCs and the precipitate was then dissolved in toluene.

##### Preparation of PMMA‐NCs Composite Films

300 mg PMMA was dissolved in toluene with a concentration of 150 mg mL^−1^ and 2 mL NCs was added into the PMMA solution under vigorous stirring. The mixture was stored under vacuum at room temperature for 2 h to remove the bubbles in it. To prepare a PMMA‐NC film, the mixed solution (1.2 mL) was cast onto the surface of a 2 cm × 2 cm quartz substrate and the film was left to dry overnight under vacuum.

##### Characterization

The TEM images and the EDS elemental mapping of perovskite NCs were characterized by a FEI Tecnai G2 F20 operating at an accelerating voltage of 200 kV equipped with a charge‐coupled device camera (Quemese, EMSIS GmbH). The absorbance spectra were measured by a UV–vis spectroscopy (Purkinje, TU‐1900). The PL spectra were recorded using an integrating sphere system equipped with a 360 nm continuous wave laser and a fiber‐coupled spectrometer (Ocean Optics, QE Pro). The absolute PLQY and time‐resolved photoluminescence were measured on an Edinburgh FLS1000 instrument (excited at 365 nm). XRD analysis was carried out on a Bruker D8 Advance diffractometer (Cu K‐alpha radiation, lambda = 1.5418 Å). XPS analysis (Physical Electronics [PHI] 5300, 250 W, 12 kV) was performed on a PHI 5300 (250 W, 12 kV) using Al as the target anode. ICP‐MS was carried out on a Thermo Fisher iCAP Q machine.

##### First Principles Calculations

First principles methods based on density functional theory (DFT)^[^ ^53^, ^54^ ^]^ with the projector‐augmented wave method (PAW)^[^ ^55^, ^56^ ^]^ were used as implemented in the Vienna ab initio simulation package (VASP).^[^ ^57^, ^58^ ^]^ The generalized gradient approximation was used in the Perdew–Burke–Ernzerhof revised for solids parametrizations of the exchange‐correlation functional.^[^ ^59^ ^]^ PAW potentials containing nine valence electrons for Cs (5s^2^5p^6^6s^1^), 14 valence electrons for Pb (5d^10^6s^2^6p^2^), and seven valence electrons for Br (4s^2^4p^5^) were employed. For Nd a valency of 3 was obtained by placing three 4f electrons in the core, and one extra f electron in the valence together with the atomic 5s^2^5p^6^6s^2^ states, making a total of eleven valence electrons, which was based on the standard model for the treatment of localized f electrons. Based on convergence tests, a plane‐wave basis set with a kinetic energy cutoff of 250 eV and a BZ grid of 6 × 6 × 6 Γ‐centered *k*‐points was used for the structural relaxation of the unit cell until the energy differences are converged within 10^−6^ eV, with a Hellman–Feynman force convergence threshold of 10^−2^ eV Å^−1^. For Nd^3+^‐doped CsPbBr_3_, the cubic phase was used as a reference structure to construct 2 × 2 × 2 and 3 × 3 × 3 supercells with uniform 3 × 3 × 3 and 2 × 2 × 2 Γ‐centered *k*‐point grids, respectively. *G*_0_ *W*_0_ corrections were also calculated on top of the DFT results, using an energy cutoff for the response function set to be 200 eV, combined with a 6 × 6 × 6 Γ‐centered *k*‐point grid as implemented in VASP.^[^ ^60^, ^61^, ^62^ ^]^ In addition, self‐consistent quasiparticle *GW* (QP*GW*) calculations were performed where *G* and *W* were iterated six times.^[^ ^63^, ^64^ ^]^ To compute the optical properties, the Kohn–Sham wavefunctions with a kinetic energy cutoff of 250 eV and a Γ‐centered *k*‐point grid of size 24 × 24 × 24 were used. A parametrized model was used for the dielectric screening function by fitting the QP*GW* result, and the DFT energy eigenvalues were shifted with a scissor operator instead of using the QP*GW* eigenvalues. Then the model Bethe–Salpeter equation (mBSE) was solved using the Tamm–Dancoff approximation with a total of 16 valence and 16 conduction bands.^[^ ^65^, ^66^ ^]^ For a 6 × 6 × 6 Γ‐centered *k*‐point grid, the mBSE method was of similar accuracy as the Bethe–Salpeter equation on top of QPGW calculations.^[^ ^67^, ^68^ ^]^ A finite‐temperature correction was also included to the bandgap arising from thermal fluctuations taken from previous work.^[^ ^69^ ^]^ Spin–orbit coupling was included throughout all electronic structure and optical properties calculations using the second‐variational method, in which the spin–orbit interaction was included as a perturbation to the scalar relativistic Hamiltonian.^[^ ^70^ ^]^

## Conflict of Interest

The authors declare no conflict of interest.

## Supporting information

Supporting Information
