# Supplementary material for: Highly Efficient Blue‐Emitting CsPbBr3 Perovskite Nanocrystals through Neodymium Doping
Source: Adv Sci (Weinh). 2020 Sep 3;7(20):2001698. doi: 10.1002/advs.202001698 (PMC7578857; doi:10.1002/advs.202001698)
Supplement: Supplementary file 1 — Supporting Information [file ADVS-7-2001698-s001.pdf]

© 2020 Wiley-VCH GmbH

ADVANCED SCIENCE
Open Access

Supporting Information

for *Adv. Sci.,* DOI: 10.1002/advs.202001698

Highly Efficient Blue-Emitting $CsPbBr_3$ Perovskite Nanocrystals through Neodymium Doping

Yujun Xie, Bo Peng, Ivona Bravi´c, Yan Yu, Yurong Dong, Rongqing Liang, Qiongrong Ou, Bartomeu Monserrat, and Shuyu Zhang**

Supporting Information

Highly Efficient Blue-Emitting $CsPbBr_3$ Perovskite Nanocrystals through Neodymium Doping

Yujun Xie, Bo Peng, Ivona Bravić, Yan Yu, Yurong Dong, Rongqing Liang, Qiongrong Ou, Bartomeu Monserrat, and Shuyu Zhang**

Summary:

Figure S1. X-ray photoelectron spectroscopy analysis of Cs^+

Figure S2. Size distribution of perovskite nanocrystals

Figure S3. Additional X-ray diffraction patterns

Figure S4. Energy dispersive X-ray spectroscopy

Figure S5. Tauc plots of $(\alpha h\nu)^2$ vs photon energy

Figure S6. Projected density of states

Figure S7. Calculated band gap correction as a function of the dopant concentration

Figure S8. Calculated exciton binding energy

Figure S9 Thermal cycling spectra

Figure S10. Photostability test

Figure S11. Additional photoluminescence spectra

Table S1. Calculated lattice constants with different phases

Table S2. ICP-MS

Table S3. Calculated band gaps data

Table S4. Detailed optical performance data

Table S5. Summary of the blue-emitting lead-halide (doped) perovskite nanocrystals

Table S6. Fitted parameters of the photoluminescence decay curves

Table S7. Calculated exciton binding energy data

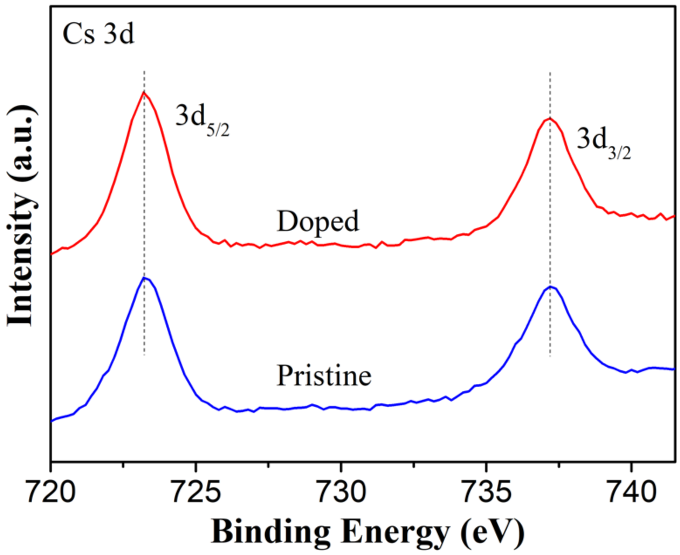

Figure S1. High-resolution X-ray photoelectron spectroscopy analysis of Cs^{+} cation. No obvious shift of the peaks is observed.

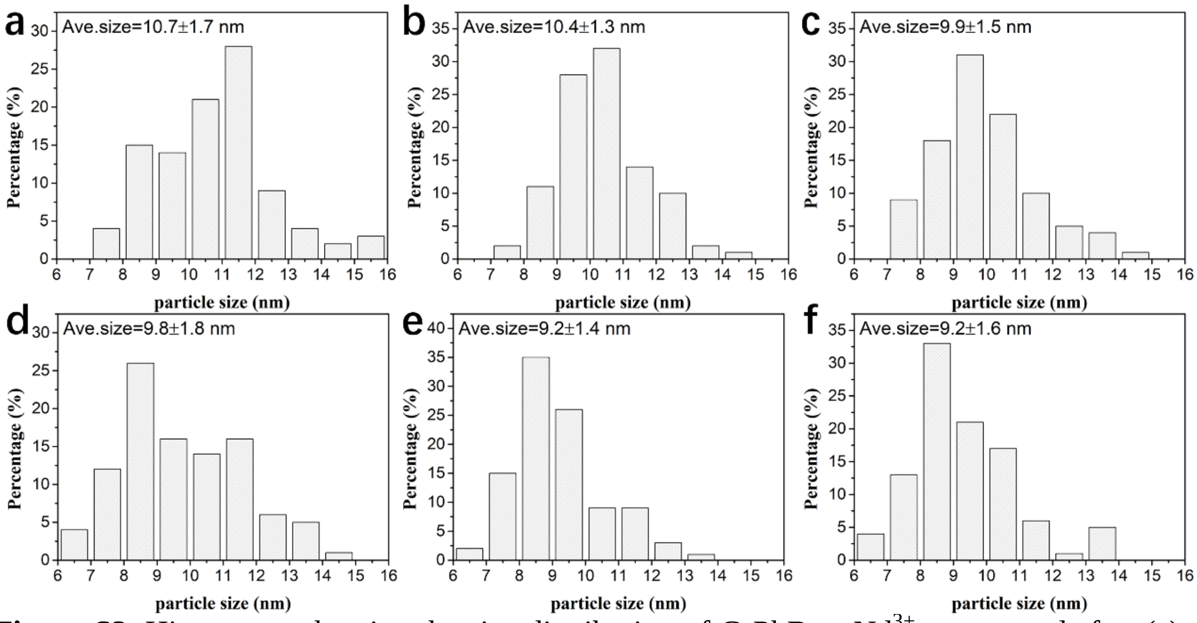

Figure S2. Histograms showing the size distribution of $CsPbBr_3$:$x$$Nd^{3+}$ nanocrystals for: (a) x = 0, (b) x = 2.7%, (c) x = 3.5%, (d) x = 6.0%, (e) x = 7.0%, (f) x = 7.2%.

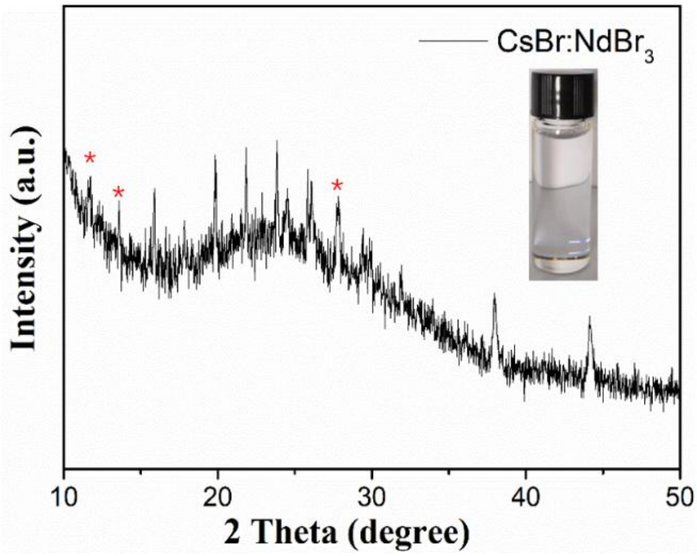

Figure S3. X-ray diffraction pattern of nanocrystals prepared using $NdBr_3$ to replace $PbBr_2$ without adding any $PbBr_2$. The inset shows the corresponding non-luminescent $CsBr:NdBr_3$ colloidal solution.

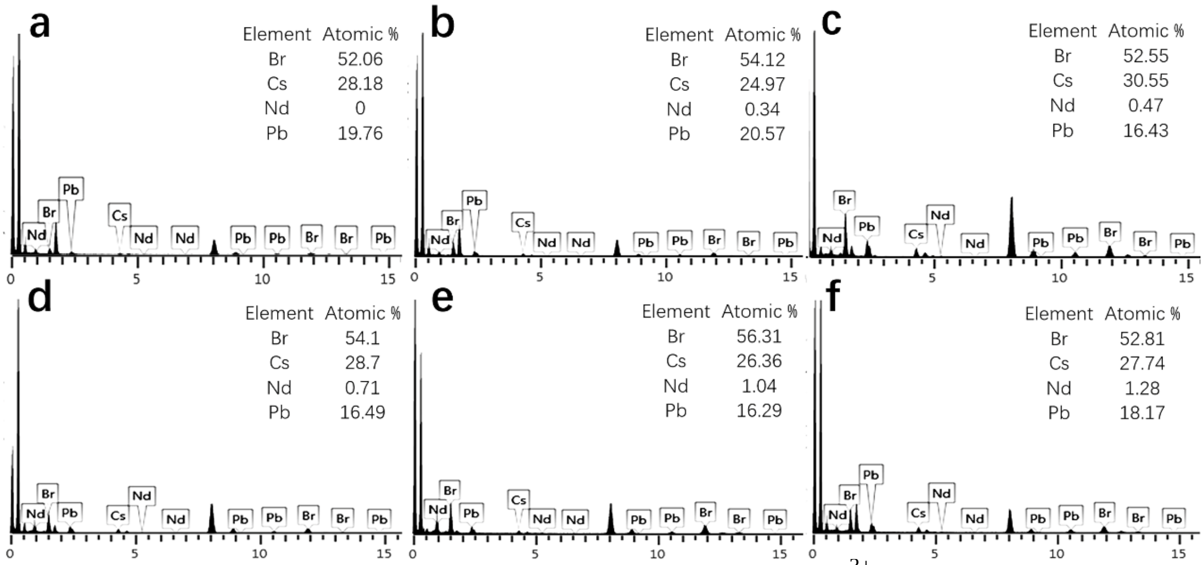

Figure S4. Energy dispersive X-ray spectroscopy of $CsPbBr_3$:$x$$Nd^{3+}$ nanocrystals for: (a) x = 0, (b) x = 2.7%, (c) x = 3.5%, (d) x = 6.0%, (e) x = 7.0%, (f) x = 7.2%.

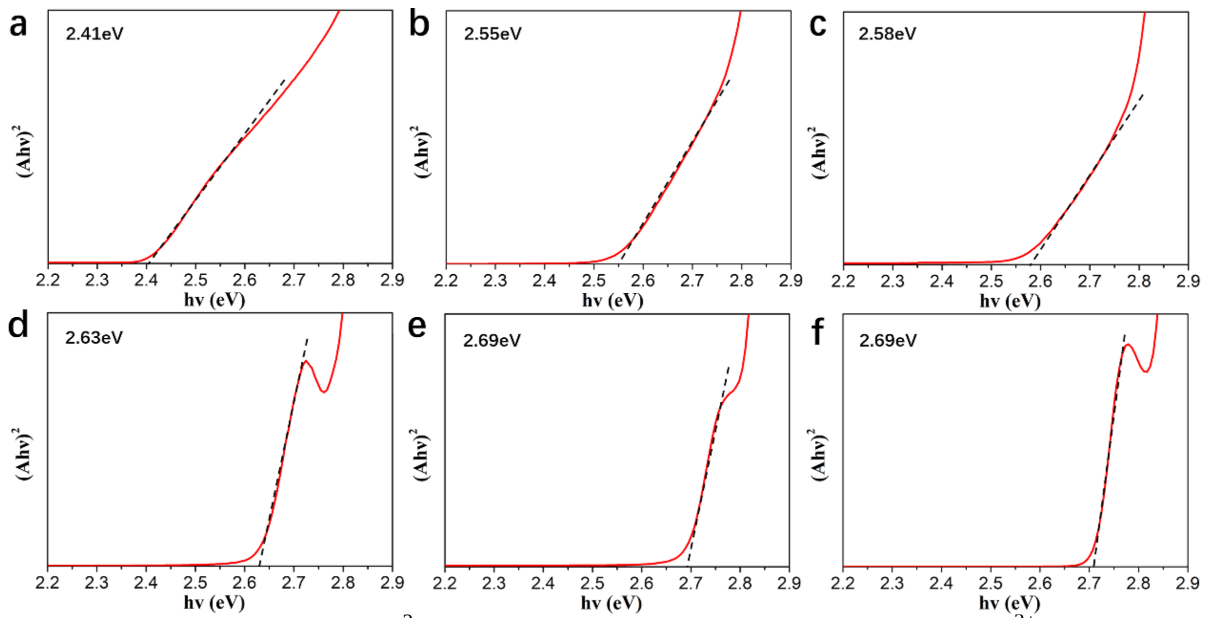

Figure S5. Tauc plots of $(\alpha h\nu)^2$ against photon energy (eV) for $CsPbBr_3$:*x*Nd^{3+} nanocrystals for: (a) *x* = 0, (b) *x* = 2.7%, (c) *x* = 3.5%, (d) *x* = 6.0%, (e) *x* = 7.0%, (f) *x* = 7.2%.

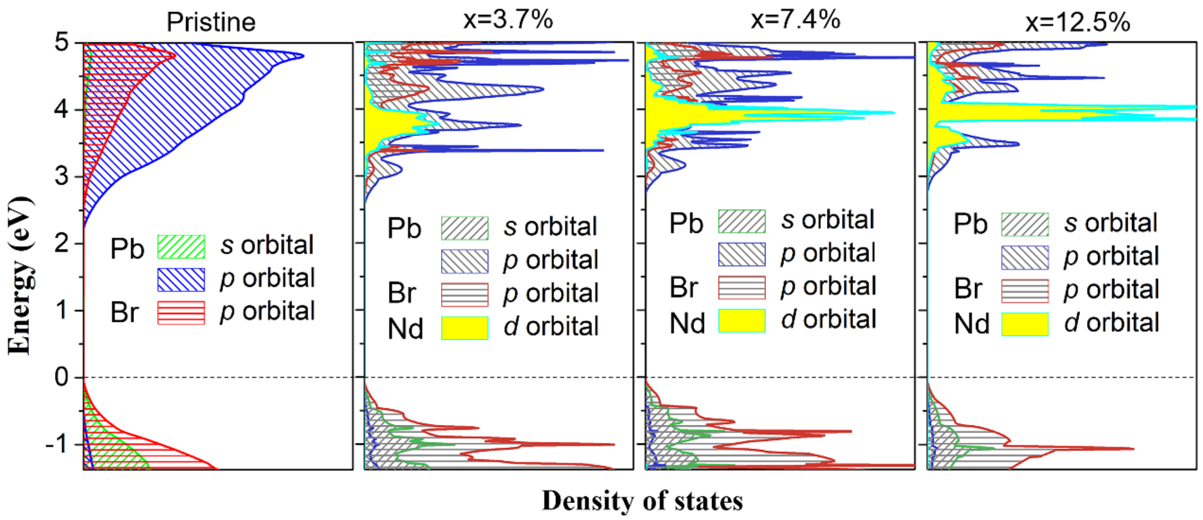

Figure S6. Calculated projected density of states of bulk cubic $CsPbBr_3$ with increasing Nd dopant ratio. The VBM is set at zero energy.

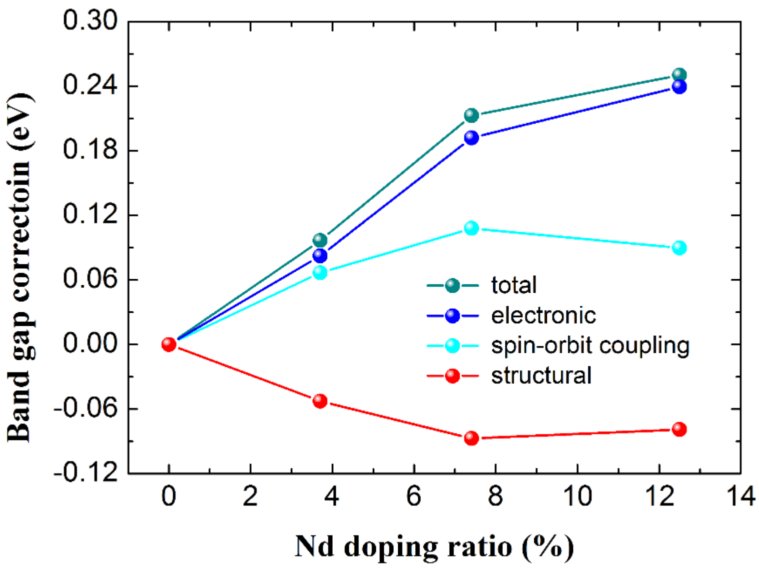

Figure S7. Calculated band gap correction as a function of the dopant concentration from individual contributions as well as the interplay between electronic effect, spin-orbit coupling and structural effects.

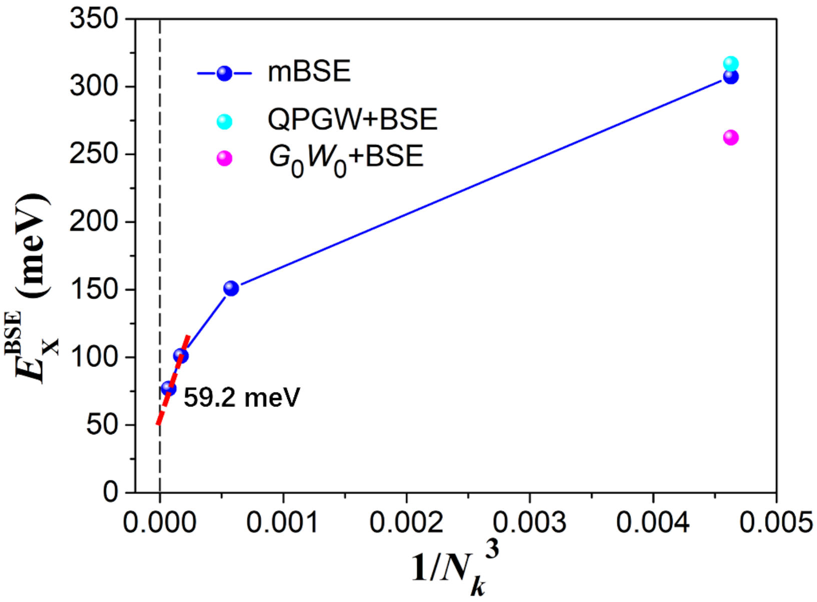

Figure S8. Binding energy of the first bright exciton as a function of **k**-point sampling grid size. The model Bethe-Salpter equation (mBSE) results are in good agreement with the QP*GW*+BSE calculation. Coarse **k**-point sampling significantly overestimates the binding energy E_x^{mBSE}, and the E_x^{mBSE} presented in Table S7 is the result of a linear extrapolation to an infinite **k**-point sampling grid, which has been widely used to determine E_x^{mBSE} [*Sci. Rep.* **2016**, 6, 1; *Phy. Rev. B* **2008**, 78, 085103].

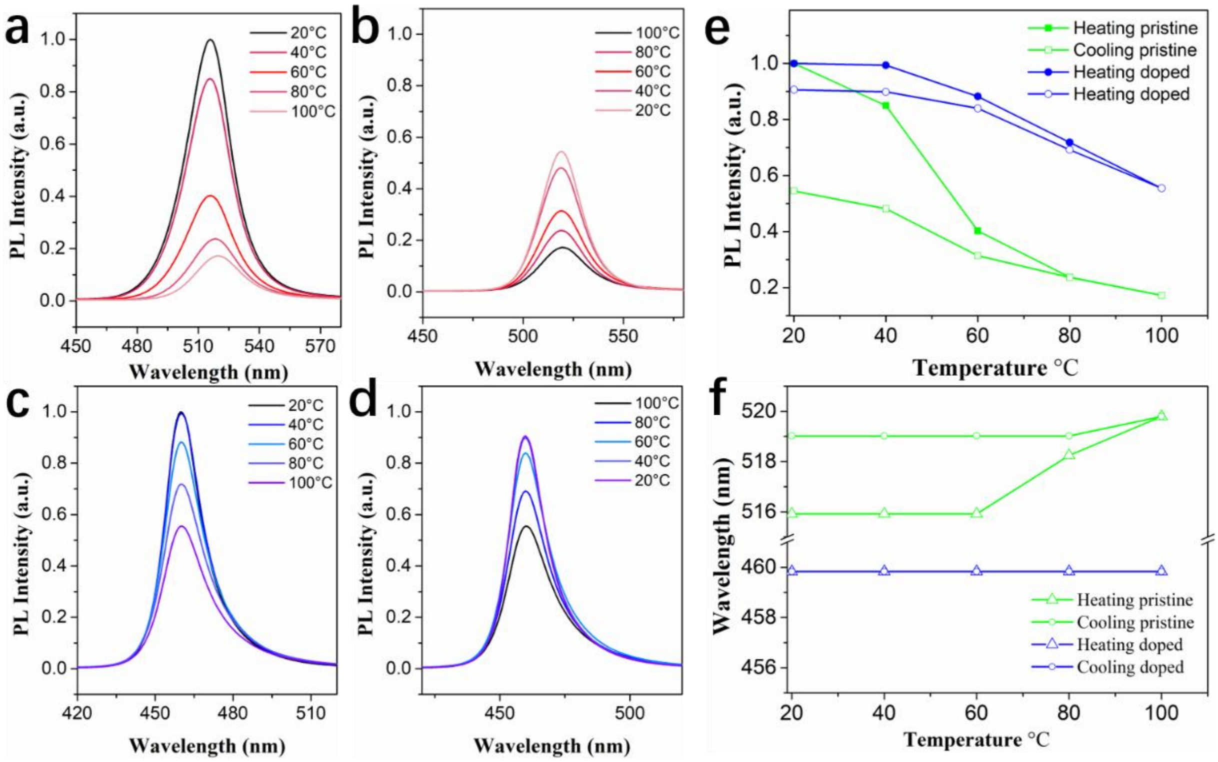

Figure S9. Thermal cycling spectra of pristine $CsPbBr_3$ nanocrystals (a, b) and $CsPbBr_3$:$x$$Nd^{3+}$ (x = 7.2%) nanocrystals (c, d). Variation of (e) the relative photoluminescence peak intensity and (f) the photoluminescence peak position in the thermal cycling test. The controlled heating ramp is 5 °C/min.

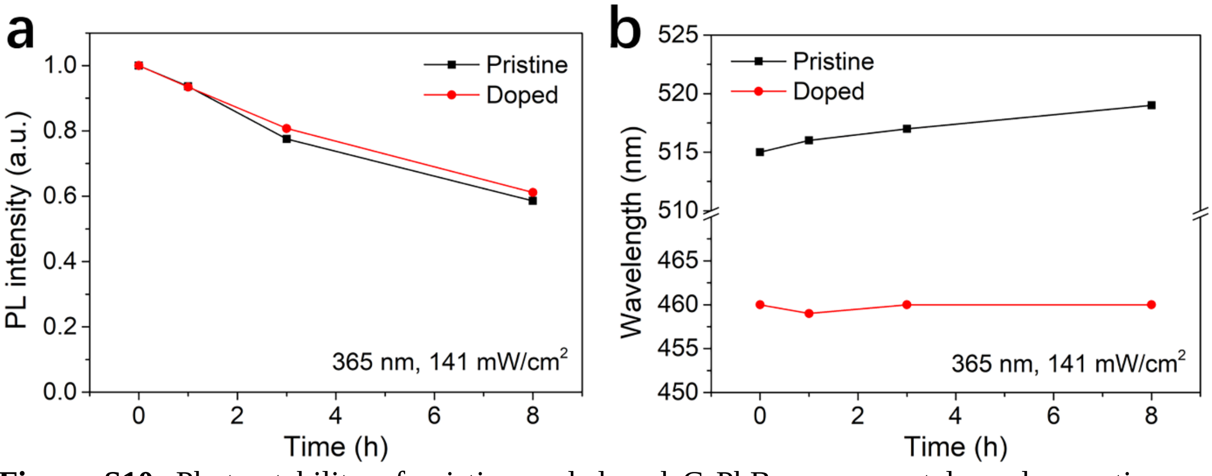

Figure S10. Photo-stability of pristine and doped $CsPbBr_3$ nanocrystals under continuous ultraviolet irradiation (365 nm, 141 mW/cm^2). Variation of (a) the relative photoluminescence peak intensity and (b) the photoluminescence peak position in the photo-stability test.

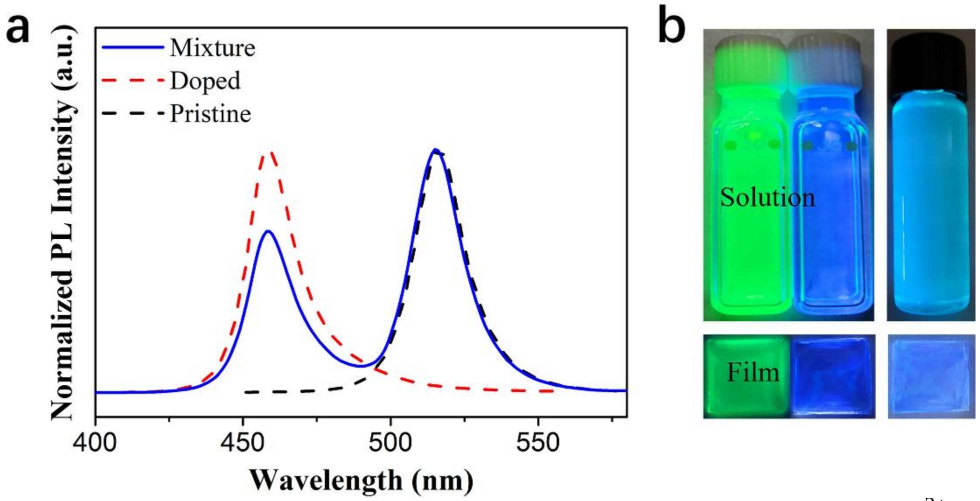

Figure S11. (a) Photoluminescence spectra of the mixture of blue $CsPbBr_3$:$x$$Nd^{3+}$ (x = 7.2%) and green $CsPbBr_3$ nanocrystals under excitation of a ultraviolet lamp (365 nm). (b) The luminescent perovskite nanocrystals in toluene (top) and encapsulated into PMMA to form a film (bottom) (left: pristine; middle: doped; right: mixed).

Table S1. Calculated bulk lattice constant and experimental nanocrystal lattice constants of cubic $CsPbBr_3$ (*Pm*-3*m* space group). The high temperature phase of $CsPbBr_3$ is cubic with space group *Pm*-3*m*. The lattice constant using the PBEsol functional is in better agreement with experiment than the value obtained with the PBE functional, and we therefore employ the PBEsol functional in all our calculations.

Space group	Samples	Methods	*a* (Å)
Pm-3*m*	$CsPbBr_3$	PBE	6.000
		PBEsol	5.869
		Exp.	5.85
	$CsPbBr_3$:$x$$Nd^{3+}$ (x = 7.4%)	PBEsol	5.823

Table S2. ICP-MS analysis of $CsPbBr_3$:$x$$Nd^{3+}$ nanocrystals.

$NdBr_3$/$PbBr_2$ mole feeding ratio	Nd (mg/L)	Pb (mg/L)	Nd (μmol/L)	Pb (μmol/L)	Nd atomic doping ratio x = Nd/(Pb+Nd)
0.3	1.21	62.25	8.42	300.70	2.7%
0.6	1.54	60.94	10.66	294.44	3.5%
0.9	1.20	27.07	8.30	130.75	6.0%
1.2	1.34	25.55	9.27	123.43	7.0%
1.5	1.73	31.93	11.99	154.25	7.2%

Table S3. Calculated band gaps of $CsPbBr_3$ using PBEsol, HSE06, G_0W_0, and QP*GW*, including spin-orbit coupling in all calculations. The PBEsol band gap of 0.33 eV is much smaller than the experimental band gap of 2.41 eV, because semilocal DFT severely underestimates single particle band gaps [*Int. J. Quantum Chem*. **1985**, 28, 497]. The HSE06 band gap increases to 0.87 eV, still significantly underestimating the experimental value. Quasiparticle corrections calculated with the G_0W_0 and QP*GW* methods further increase the band gap, but still underestimate the experimental value of 2.41 eV. Previous work has shown that band gap corrections induced by thermal fluctuations induce a blue shift in halide perovskites, and we use a finite temperature correction Δ_T of 0.45±0.06 eV taken from *J. Phy. Chem. Lett.* **2017**, 8, 5507. The band gap is thus estimated to be $E_g^{\mathrm{total}} = E_g^{\mathrm{QP}GW} + \Delta_T$ = 2.34±0.06 eV, in good agreement with the measured band gap [*Chem. Mater. **2017***, 29, 3644]. Due to quantum confinement in nanocrystals, which is being neglected in our first principles bulk calculations, the measured photoluminescence peak increases to 2.41 eV.

Space group	PBEsol (eV)	HSE06 (eV)	G_0W_0 (eV)	QP*GW* (eV)	Δ_T (eV)	E_g^{total} (eV)
Pm-3*m*	0.33	0.87	1.16	1.89	0.45±0.06	2.34±0.06

Table S4. Optical performance of the $CsPbBr_3$:*x*Nd^{3+} NCs solution.

x	PLQY (%)	FWHM (nm)	Peak (nm)
0	81	21	515
2.7%	78	22	494
3.5%	75	24	484
6.0%	84	23	472
7.0%	89	19	465
7.2%	90	19	459

Table S5. A summary of the optical properties of a range of blue-emitting lead-halide perovskite nanocrystals. The references are: [1] *Adv. Mater.* **2016,** 28, 9163, [2] *Adv. Funct. Mater.* **2016,** 26, 2584, [3] *Adv. Sci.* **2017,** 4, 1700335, [4] *Adv. Opt. Mater.* **2019,** 7, 1901440, [5] *J. Am. Chem. Soc.* **2017,** 139, 4087, [6] *J. Phys. Chem. Lett.* **2019**, 10, 943, [7] *Nanoscale,* **2020**, 12, 11728.

Samples	PL Peak position (nm)	FWHM (nm)	PLQY (%)	PL Lifetime (ns)	Reference
$MAPbCl_{0.5}Br_{2.5}$	458	29	38.4	--	[1]
$CsPbCl_{1.5}$ $Br_{1.5}$	455	16	37	--	[2]
$CsPbBr_3$:Al^{3+}	456	16	42	14	[3]
Rb^+:$CsPbBr_3$	465	18	60	5.6	[4]
$CsPbBr_3$:Cd^{2+}	452	≈16	≈60	6.4	[5]
$CsPbBr_3$:Zn^{2+}	462	≈16	≈60	7.2	
$CsPb_{0.93}Cu_{0.07}(Br/Cl)_3$	455	23	80	4.2	[6]
PEA-$CsPb(Cl/Br)_3$	470	21	80	--	[7]
$CsPbBr_3$:*x*Nd^{3+} (*x* = 7.2%)	459	19	90	5.27	This work

Table S6. Fitted parameters of the decay curves of $CsPbBr_3$:*x*Nd^{3+} nanocrystals. τ_{ave}, k_r and k_{nr} are calculated according to the following equations [*Principles of fluorescence spectroscopy*, 3rd ed.; Springer: New York, 2006; pp 8-10, 99, 141-143.]: (i) the average photoluminescence lifetimes: $\tau_{ave} = \frac{□_1 * □_1^2 + □_2 * □_2^2}{□_1 * □_1 + □_2 * □_2}$, (ii) the radiative lifetime τ_r and nonradiative lifetime τ_{nr}: $\frac{1}{□_□} + \frac{1}{□_{□□}} = \frac{1}{□_{□□□}}$, (iii) the radiative decay rate: $k_r = \frac{1}{□_□}$, (iv) the nonradiative decay rate: $k_{nr} = \frac{1}{\tau_{nr}}$, and (v) the photoluminescence quantum yield: PLQY = $\frac{\frac{1}{\tau_r}}{\frac{1}{\tau_r}+\frac{1}{\tau_{nr}}}$.

x	A_1 (%)	τ_1 (ns)	A_2 (%)	τ_2 (ns)	τ_{ave} (ns)	k_r ($\times 10^7 s^{-1}$)	k_{nr} ($\times 10^7 s^{-1}$)
0	83.5	4.34	16.5	23.32	14.11	5.77	1.32
2.7%	82.5	4.57	17.5	18.53	11.03	7.07	2.00
3.5%	82.1	4.41	17.9	16.81	10.04	7.47	2.49
6.0%	86.2	4.32	13.8	14.15	7.70	10.97	2.02
7.0%	73.5	4.11	26.5	8.98	6.26	14.25	1.73
7.2%	50.4	3.18	49.6	6.34	5.27	17.11	1.85

Table S7. Effective electron and hole masses, high-frequency dielectric constants, and the Wannier-Mott exciton binding energies for pristine and doped $CsPbBr_3$:$x$$Nd^{3+}$ (x = 12.5%). The mBSE binding energy for the pristine case is also listed for comparison. In the Wannier-Mott model for electron-hole pairs interacting through a screened Coulomb potential in parabolic bands, the exciton binding energy is $E_x^{WM} = (\mu/\varepsilon_\infty^2)\,R$, where μ is the effective mass of the electron-hole pair $\mu^{-1} = m_h^{-1} + m_e^{-1}$, ε_∞ is the high-frequency dielectric constant, and R is the Rydberg constant. The high-frequency dielectric constant is calculated using density functional perturbation theory [*Phy. Rev. B* **2005**, 72, 035105] as implemented in VASP. This approach has been widely used to study exciton binding energies in perovskites [*Sci. Rep.* **2016**, 6, 1; *Chem. Mater.* **2017**, 29, 524]. The mBSE binding energy of 59.2 meV agrees well with the Wannier-Mott estimate of the exciton binding energy.

Samples	m_h (m_0)	m_e (m_0)	ε_∞	E_x^{WM} (meV)	E_x^{mBSE} (meV)
Pristine	0.206	0.213	4.699	64.6	59.2
12.5% doped	0.329	0.345	4.932	94.3	-
